# Dose‐dependent effects of aerobic exercise on clinically relevant biomarkers among healthy women at high genetic risk for breast cancer: A secondary analysis of a randomized controlled study

**DOI:** 10.1002/cnr2.1497

**Published:** 2021-07-09

**Authors:** Christopher J. Ehret, Shouhao Zhou, Julia C. Tchou, Kathryn H. Schmitz, Kathleen M. Sturgeon

**Affiliations:** ^1^ Department of Public Health Sciences, College of Medicine Pennsylvania State University Hershey Pennsylvania USA; ^2^ Department of Surgery, Division of Endocrine and Oncologic Surgery, Perelman School of Medicine University of Pennsylvania Philadelphia Pennsylvania USA

**Keywords:** aerobic capacity, aerobic exercise, biomarkers, BRCA, breast cancer, high‐risk, VO_2_max

## Abstract

**Background:**

Excess adiposity and dysregulated metabolism are associated with increased cancer risk. Triglycerides, cholesterol, glucose, insulin, HOMA‐IR, and VO_2_max are robust clinical‐metabolic biomarkers of overall health.

**Aims:**

Aerobic exercise may improve clinical‐metabolic biomarkers and decrease cancer risk. This secondary analysis of the WISER Sister randomized controlled trial investigated dose‐dependent effects of aerobic exercise on clinical biomarker levels in women at high genetic risk for breast cancer.

**Methods and Results:**

One hundred thirty‐nine participants were randomized to: control (<75 min/week), low‐dose (150 min/week), and high‐dose (300 min/week) aerobic exercise intervention groups. Intervention adherence was assessed via heart monitor. Fasting blood draws, cardio‐pulmonary tests, and demographical surveys were taken at baseline and 5 months. Triglyceride, cholesterol, glucose, insulin, and VO_2_max changes were assessed for 80 of the 122 study completers. Ninety‐six percent of assayed‐completers adhered to >80% of their exercise dose. A significant dose‐dependent increase in VO_2_max was observed for the low‐dose and high‐dose groups compared to control. No intervention effects were observed for plasma biomarkers. Overweight women (BMI > 25) showed a significant decrease in insulin levels and a trend for decreased triglycerides following exercise intervention. Significant increases in VO_2_max were independent of BMI stratification.

**Conclusion:**

Women at high genetic risk for breast cancer should maintain healthy weights and aerobic capacities through aerobic exercise to achieve measurable benefits on overall health. For overweight women, exercise appears to improve subclinical metabolic dysregulation. However, normal weight women were unaffected by aerobic exercise as their biomarker levels may be below the threshold for improvement. VO_2_max increases solely quantified the benefits of exercise in already healthy women at high‐risk for breast cancer.

## 1 INTRODUCTION

Genome‐wide association studies have identified rare coding and noncoding mutations that influence the risk for developing breast cancer.^1^ More specifically, variants of genes involved in DNA repair, such as BRCA 1/2 (germline), p53 (somatic), PTEN (germline), CHEK2 (germline), and PALB2 (germline), have been closely associated with significantly increased risk of breast cancer development.^2^, ^3^ Heterogeneity at the genetic, molecular, and environmental levels, make preventing breast cancer difficult.^4^ In addition to genomics and environment, lifestyle factors play a key role in the development and growth of breast cancers. The worldwide obesity epidemic has proven to be a pivotal influencer on increased cancer risk and worsening outcomes in cancer treatment. Moreover, systemic metabolic dysregulation (including hyperglycemia, insulin resistance/hyperinsulinemia, and dyslipidemia) found within the obesogenic milieu can increase the growth and development of many cancers.^5^, ^6^ Specifically, this metabolic dysregulation can decrease cancer cell apoptosis and increase cancer cell proliferation and angiogenesis.^6^, ^7^, ^8^, ^9^

Physical activity has been shown to reduce breast cancer risk in women.^10^, ^11^, ^12^ Women at a high genetic risk (≥18%) for developing breast cancer (BRCA 1/2 carriers) have been evaluated to determine the effects of physical activity on breast cancer risk.^13^, ^14^ Reduced breast cancer rates in female BRCA 1/2 carriers partaking in physical activity were observed.^13^, ^14^ Recent studies have also shown favorable dose‐dependent effects of aerobic exercise on factors contributing to breast cancer risk, such as breast density, hormone levels, body composition, and adipokines in females at high genetic risk for developing breast cancer.^15^, ^16^, ^17^, ^18^ Although these studies have shown that physical activity can decrease breast cancer risk, a randomized clinical trial assessing the effects of aerobic exercise on cancer‐related clinical‐metabolic biomarkers in a dose‐dependent manner does not exist. Therefore, the objective of this secondary analysis was to evaluate dose‐dependent effects of aerobic exercise on cancer‐linked clinical‐metabolic biomarkers in healthy, sedentary, eumenorrheic women at high genetic risk for developing breast cancer whom participated in the WISER (Women in Steady Exercise Research) Sister Study.

Robust, clinically relevant biomarkers of overall health related to metabolic dysfunction were examined (VO_2_max, BMI, triglycerides, cholesterol, glucose, and insulin).^19^ We sought to investigate the dose‐dependent effects of aerobic exercise on these clinical‐metabolic biomarkers in a rare population of healthy females at high genetic risk for developing breast cancer stratified by BMI. An accurate and dependable biomarker for evaluating aerobic exercise in this population would provide an opportunity to closely monitor exercise dosing, as aerobic exercise may delay the onset of breast cancer in these healthy, high‐risk females.

## 2 MATERIALS AND METHODS

### 2.1 Participant eligibility, recruitment, and randomization

**FIGURE 1:**
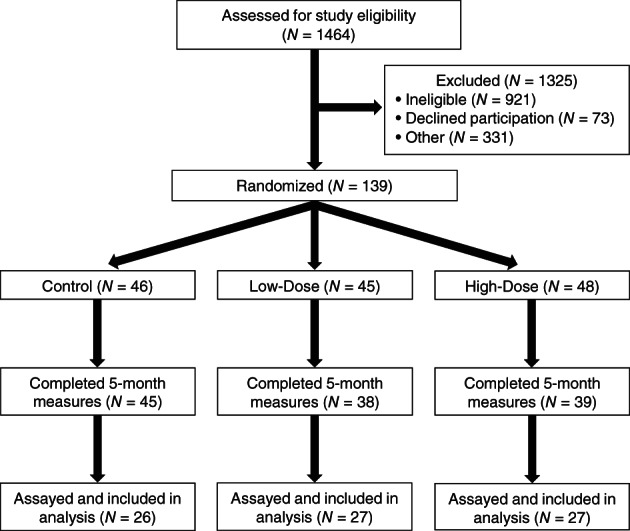
CONSORT diagram of participant flow throughout study

The WISER Sister study was a randomized, controlled, three‐group, parallel‐arm study conducted from 2008 to 2012, evaluating the physiologic effects of low and high dose aerobic exercise in healthy, sedentary, eumenorrheic women at elevated risk for breast cancer. Participants were recruited across the United States using national organizations such as the Cancer Genetics Network and Facing Our Risk of Cancer Empowered.^20^ Eligibility screening via telephone identified healthy, sedentary (≤75 min of exercise per week), eumenorrheic, women aged 18 and older who were nonsmokers, with a BMI ranging from 18 to 50 kg/m^2^. These women were selected if they were at increased risk for developing breast cancer, defined as ≥18% life time risk for developing breast cancer via (1) Claus prediction models, (2) known BRCA1/2 mutations, or (3) known family members with a deleterious mutation conferring a ≥25% probability of a deleterious mutation in the participant.^21^, ^22^ Of the 1464 women contacted, 1133 were screened, 217 were eligible, 162 were consented, 139 were randomized, 122 completed the study. The first 80 of these 122 completers were assayed, as shown in Figure 1. Women were equally randomized to control (≤75 min/week), low‐dose (150 min/week), or high‐dose (300 min/week) intervention groups after stratification by (1) years since starting menstruation (≤10 vs. >10 years) and (2) BMI (<30 vs. 30 kg/m^2^). The study was approved by the University of Pennsylvania Institutional Review Board, and written consent was obtained prior to participation by all participants and sent to their respective physicians. Additional details about eligibility and recruitment has been previously published. (NCT ClinicalTrials.gov registration #: NCT00892515).^15^

### 2.2 Exercise intervention

Participants in the low and high‐dose intervention groups completed their prescribed aerobic exercise interventions over approximately 5 months (five menstrual cycles) using study‐provided treadmills in their homes. Exercise intensity remained the same between intervention groups and was set at 65%–70% of age‐predicted maximum heart rate for the first month, which was then increased to 70%–80% for the remainder of the study. The duration of exercise for the high‐dose intervention group increased after the first month (150 min/week) via intervals of 20–25 min every 2 weeks until reaching 300 min/week by week 11. Of note, only exercise completed within the target percent range of age‐predicted maximum heart rate was counted toward the total minutes of exercise for each week. To monitor exercise adherence, women in the intervention groups kept exercise logs, wore heart monitors (U.S. model RS400, Polar Electro Inc., Lake Success, NY), and transmitted or presented their weekly data to study staff for review. Exercise adherence data including total time at target heart‐rate range and percent of prescribed time completed was collected weekly and the total data collected at the end of the study. Those in the control group were instructed to continue their current, prestudy exercise regimens (≤75 min/week) and refrain from starting new forms of exercise throughout the duration of the study. Strength and flexibility training were not included in the exercise restrictions for the control group. Additional details about the exercise intervention has been previously published.^15^

### 2.3 Data collection

Women provided demographic characteristics at the beginning of the study. Blood draws were completed at baseline (6–10 days after start of menstrual cycle) and approximately 5 months later at follow‐up (6–10 days after start of menstrual cycle). Samples were stored at −80°C and a random subset of samples from women whom completed the study were assayed for triglyceride, cholesterol, glucose, and insulin levels. Body weight and height was recorded at baseline and follow‐up, and used to calculate BMI. Participants' aerobic fitness level was assessed at baseline and follow‐up using a maximal treadmill test, the Bruce protocol, and a 6–20 perceived exertion scale.^23^, ^24^ VO_2_max was estimated using the method by Pollock et al. for maximal exercise testing with the Bruce Protocol (VO_2_max = 0.073 [time in seconds on the Bruce Protocol] – 3.9).^24^ Additional details about data collection has been previously published.^15^

### 2.4 Blood assays

Total cholesterol, triglyceride, and glucose were measured on a Roche COBAS c501 clinical auto‐analyzer in single determinations. Insulin was measured by double antibody RIA HI‐14k (EMD Millipore, Billerica, MA) in duplicate. Insulin assay CVs: intra‐assay 4.99%, inter‐assay 11.3%. All assays were conducted at the Radioimmunoassay and Biomarkers Core at the University of Pennsylvania.

### 2.5 Statistical analyses

Demographic characteristics were reported for women whom completed the study and were included in the biomarker analysis (*N* = 80). Demographic characteristics were also stratified by intervention group (control: *N* = 26, low‐dose: *N* = 27, high‐dose: *N* = 27). Utilizing the Fischer exact test, categorical demographic characteristics were compared between the control and intervention groups for those women included in the biomarker analysis. The same comparison was made for continuous demographic characteristics using two‐sample *t*‐tests. Baseline clinical biomarkers were compared using global *F*‐test for analysis of variance (ANOVA). Given any p‐value <.05, further pairwise comparisons between intervention groups were adjusted with Bonferroni correction. Paired t‐tests were used to assess changes between baseline and follow‐up for clinical biomarkers within each intervention group. Absolute change in clinical biomarkers following the intervention period was evaluated via baseline‐adjusted linear regression models. Patients were stratified by baseline BMI (<25 and ≥ 25 kg/m^2^) and intervention group differences were assessed using 3 × 2 ANOVA. All parametric tests for continuous variables were run using log‐adjusted values. Statistical significance was set to an alpha level of 0.05, and all statistical analyses were completed using STATA version 12.1 (Stata Corp).

## 3 RESULTS

Demographic characteristics for those women who completed the study and had their blood assayed (*N* = 80) are presented in Table 1. There were no demographic differences at baseline between our control and intervention groups. Throughout the study, 96% of patients in the intervention groups adhered to >80% of their exercise dose.

**TABLE 1:** Demographic characteristics for WISER Sister participants

Characteristic	Overall (*N* = 80)	Control (*N* = 26)	Low (*N* = 27)	High (*N* = 27)
Age, year	35.3 ± 6.3	35.6 ± 6.7	36.5 ± 5.5	33.8 ± 6.5
Race				
White	77 (96.3%)	25 (96.2%)	27 (100%)	25 (92.6%)
Other	3 (3.8%)	1 (3.8%)	0 (0%)	2 (7.4%)
Education				
≤High school	2 (2.5%)	2 (7.7%)	0 (0%)	0 (0%)
Some college	15 (18.8%)	4 (15.4%)	5 (18.5%)	6 (22.2%)
≥College	63 (78.8%)	20 (76.9%)	22 (81.5%)	21 (77.8%)
Employed full time (% yes)	46 (57.5%)	16 (61.5%)	16 (59.3%)	14 (51.9%)
Household income				
<$20 000	2 (2.5%)	1 (3.8%)	0 (0%)	1 (3.7%)
$20000–39 999	9 (11.3%)	3 (11.5%)	2 (7.4%)	4 (14.8%)
≥$40 000	66 (82.5%)	21 (80.8%)	24 (88.9%)	21 (77.8%)
Marital status				
Single/divorced/separated	24 (30%)	13 (50.0%)	2 (7%)	9 (33.3%)
Married/partnered	56 (70.0%)	13 (50.0%)	25 (93%)	18 (66.7%)
Children (% yes)	51 (63.8%)	14 (53.8%)	23 (85%)	14 (51.9%)
Age at first birth, year	28.3 ± 3.9	27.7 ± 4.7	28.4 ± 3.8	28.7 ± 3.7
Age at Menarche, year	12.5 ± 1.3	12.9 ± 1.5	12.3 ± 1.3	12.4 ± 1.2
Alcohol intake, glass	0.3 ± 0.4	0.3 ± 0.4	0.4 ± 0.6	0.4 ± 0.3
Adherence	97.0 ± 11.9	‐	99.6 ± 12.3	94.4 ± 11.1
BMI, kg/m^2^	25.9 ± 5.8	27.5 ± 6.6	25.9 ± 5.4	24.2 ± 4.9

*Note*: Values are mean ± SD or *N* (%).

**FIGURE 2:**
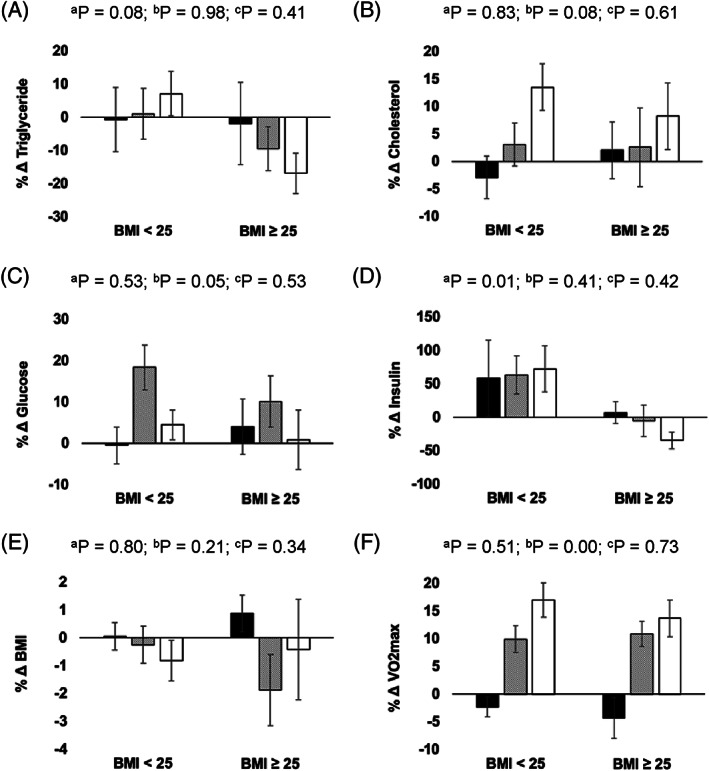
Percent change in (A) triglycerides, (B) total cholesterol, (C) glucose, (D) insulin, (E) body mass index (BMI), and (F) VO_2_max from baseline to follow‐up, stratified by baseline BMI and intervention arm. Control group = black bars, Low‐dose exercise (150 min/week) = hash‐marked bars, High‐dose exercise (300 min/week) = white bars. ^a^ BMI main effect; ^b^ Exercise intervention main effect; ^c^ Interaction effect. Mean ± SEM presented

Clinical biomarker data for all treatment groups are found in Table 2. At baseline, a significant difference in mean cholesterol levels was appreciated between high‐dose and control groups. There were no changes between treatment groups in metabolic biomarker levels following the intervention. A significant dose‐dependent increase in VO_2_max was observed for the low‐dose and high‐dose groups, as compared to the control. Figure 2 shows clinical biomarker percent change means and standard error of means (avg. %∆ ± SEM), stratified by BMI utilizing the overweight BMI cutoffs of <25 kg/m^2^ (control: *N* = 14, low‐dose: *N* = 14, high‐dose: *N* = 18) and ≥25 kg/m^2^ (control: *N* = 12, low‐dose: *N* = 13, high‐dose: *N* = 9). There were no BMI or exercise effects on the change in triglycerides for women depending on their baseline BMI (Figure 2(A)). This was also true for percent change in cholesterol (Figure 2(B)). There was a significant exercise effect (p = .05) for an increase in glucose levels independent of BMI and this was largely seen in the low dose exercise group (Figure 2(C)). There was a significant BMI effect (p = .01) following the exercise intervention as insulin levels were significantly increased in normal weight women for all exercise groups compared to overweight women (Figure 2(D)). There were no BMI or exercise effects on the change in weight for women depending on their baseline BMI (Figure 2(E)). Aerobic capacity improved following exercise (p < .001), independent of BMI (Figure 2(F)). There were no interaction effects between BMI and intervention groups for all six clinically relevant biomarkers of health.

**TABLE 2:** Clinical biomarker baseline and follow‐up data for assayed completers

Characteristic	Control (*N* = 26)		Low (*N* = 27)		High (*N* = 27)	
	Baseline	Follow‐up	Baseline	Follow‐up	Baseline	Follow‐up
Triglyceride, mg/dL	107.0 ± 59.9	97.7 ± 54.8	79.4 ± 23.4	73.3 ± 23.0	75.8 ± 30.7	70.6 ± 18.9
Cholesterol, mg/dL	203.5 ± 38.7	198.6 ± 31.4	194.0 ± 35.3	196.0 ± 38.4	177.0 ± 24.2^a^	195.6 ± 28.1^b^
Glucose, mg/dL	84.7 ± 23.3	85.2 ± 23.8	77.5 ± 11.8	87.5 ± 16.3^b^	82.9 ± 17.5	83.2 ± 9.4
Insulin μIU/mL	5.1 ± 3.3	5.6 ± 4.3	4.1 ± 2.6	4.5 ± 4.7	4.3 ± 2.7	4.3 ± 2.9
HOMA‐IR	1.1 ± 1.0	1.2 ± 1.1	0.8 ± 0.6	1.0 ± 1.1	0.9 ± 0.6	0.9 ± 0.7
BMI kg/m^2^	27.5 ± 6.6	27.7 ± 6.9	25.9 ± 5.4	25.7 ± 5.5	24.2 ± 4.9	24.1 ± 5.3
VO_2_Max mL/kg/min	32.4 ± 7.5	31.3 ± 7.9	33.0 ± 5.9	36.4 ± 6.6^b^ ^,^ ^c^	35.2 ± 6.8	40.4 ± 7.3^b^ ^,^ ^c^

Abbreviation: SD, Standard deviation.

^a^ Significantly different from control at baseline (between‐treatment group), p < .05 ➔ ANOVA.

^b^ Significantly different from baseline (within‐treatment group), p < .05 ➔ Paired *t*‐test.

^c^ Significantly different from control at follow‐up, (between treatment group, baseline adjusted), p < .05 ➔ Linear regression model.

## 4 DISCUSSION

Clinical‐metabolic biomarkers such as total cholesterol, triglycerides, insulin, and glucose, have traditionally served as indicators of overall health, and these biomarkers have also demonstrated responsiveness to exercise training.^25^, ^26^, ^27^ Yet, our exercise intervention groups did not experience statistically significant, dose‐dependent improvements in cholesterol, triglycerides, glucose, insulin, or HOMA‐IR following our 5‐month study.

HOMA‐IR levels in our study indicate that our population was insulin‐sensitive and of normal glycemic control. The overall lack of change in HOMA‐IR and fasting glucose levels following an exercise training intervention is similar to observations from the HERITAGE Family study.^28^ Fasting glucose levels did demonstrate an exercise effect though, and both normal weight and overweight women in the low‐dose exercise group had the greatest increase in glucose levels. As exercise duration was the only difference between exercise intervention groups we can eliminate differences in exercise intensity as a source for this variation between intervention groups.^29^, ^30^

In our study, insulin levels also did not decrease following either exercise intervention. These findings are in opposition to the HERTIAGE Family study, where they observed an 11.2% decrease in insulin levels following 150 min/week of aerobic exercise training at 75% of VO_2_max for 20 weeks.^28^ On further examination, we observed fasting insulin levels increased in all groups (including control) for normal weight women, while overweight women decreased insulin levels with exercise training. Overweight women in our study had higher baseline insulin levels which may have allowed beneficial adaptation to the exercise interventions while normal weight women in the exercise groups had percent changes in their insulin levels similar to the control group. Lastly, timing of blood collection for fasted insulin and fasted glucose levels may impact our results. While all participants were asked to not exercise within 48 h of their final testing visit, Boulé et al. observed acute responses of fasting insulin and fasting glucose lasting up to 72 h after an exercise bout.^31^

On average we observed no change in triglyceride levels following either exercise intervention. Yet, similar to insulin levels, the basal state of our participants likely impacted the magnitude of adaptation to exercise training. The effect of exercise training on improving triglyceride levels in overweight and obese individuals is well documented.^32^ The average percent increase in total cholesterol levels from baseline to follow‐up for our high‐dose group was unexpected and is challenging to explain as we did not independently measure LDL or HDL levels. The expected contributions of exercise on lower LDL and higher HDL levels in these lipoprotein levels on total cholesterol levels is well known, and therefore we cannot accurately explain this increase in total cholesterol. Of note, the only biomarker to demonstrate a dose‐dependent improvement was VO_2_max.

In our study population of healthy women at high genetic risk for breast cancer, VO_2_max increased for all treatment groups regardless of BMI. The Fitness Registry and the Importance of Exercise: A National Data Base (FRIEND) study, was a multi‐institutional initiative focused on establishing normative cardiorespiratory fitness values (VO_2_max) in the United States throughout the adult lifespan.^33^ After comparing our cohort's VO_2_max values to the standardized values of the FRIEND trial, we found the following: all groups of our healthy and sedentary participants had VO_2_max values that averaged between the 50th and 75th percentiles for their respective age group (30–39 years).^33^ As expected, the average VO_2_max for our control group saw no improvement in their average VO_2_max values, and therefore remained in the 50–75th percentile range. Those in the low‐ and high‐dose groups experienced significant improvements in their VO_2_max values, resulting in a mean VO_2_max within the 75–90th percentile reference range.^33^ Of note, small improvements in VO_2_max have been associated with reduced all‐cause and disease‐specific mortality in healthy populations.^34^, ^35^, ^36^, ^37^ Specifically, Imboden et al conducted a longitudinal study (up to 17.7 years) in 833 participants to evaluate the changes in all‐cause and disease‐specific mortality risk reductions with improvements in cardiorespiratory fitness (VO_2_max). They reported that each 1 ml/kg/min increase in VO_2_max was associated with a 16% reduction in cancer mortality.^37^

Considering our VO_2_max findings, the lack of changes in traditional biomarkers of health for our specific population, and VO_2_max's inverse association with cancer mortality, VO_2_max may be a more relevant biomarker for assessing our rare population's biological response to a well‐controlled exercise intervention. VO_2_max as a biomarker in this sense for an apparently healthy population at high genetic risk for cancer should be noted, as this finding can translate clinically. Indeed, levels of traditional biomarkers of health have been validated in pathological populations and suggest normal and abnormal levels. However, improvements in levels of traditional biomarkers may be difficult to appreciate in already healthy patients. Moreover, healthy women at high genetic risk for breast cancer may still seek metrics to accurately assess their exercise‐based cancer prevention efforts, a role that can potentially be filled by VO_2_max.

The high adherence of our participants to their exercise interventions was a considerable strength for this study. By allowing participants to complete exercise sessions between 10 and 75 min in length, we increased behavioral acceptability, which aided attainment of weekly exercise goals.^15^ In addition, this study recruited healthy, eumenorrheic, women at high genetic risk for breast cancer nationally, improving the generalizability of our population throughout the United States. Unfortunately, despite a national cohort, our study population was overwhelming white, well‐educated, and of healthy BMI, which concomitantly also limits the generalizability of our findings. Data collection was completed by trained staff who were blinded to intervention group designations.^15^

As our analysis of the WISER Sister study was secondary to the primary outcome, we were not priori powered to detect significant dose‐dependent exercise‐induced changes in traditional biomarkers of health. This limitation may be contributing to the overall lack of significant dose‐dependent changes in these biomarkers, previously proven to improve with aerobic exercise by higher‐powered studies with similar exercise regimens. Additionally, genetic, environmental, and demographic characteristics introduce significant variability in response to exercise training and this may have been why we did not observe exercise‐induced group changes in traditional metabolic biomarkers to the dosed exercise interventions.^38^ As stated earlier, our population was healthy at baseline, and this may also be contributing to the lack of improvement in traditional biomarkers following the exercise interventions.

In conclusion, our unique population of healthy, eumenorrheic females at high genetic risk for developing breast cancer demonstrated a significant increase in VO_2_max independent of basal states such as excess weight (BMI ≥25 kg/m^2^). We observed that healthy women of normal weight did not show improvements in traditional biomarker levels such as triglycerides, total cholesterol, glucose, and insulin following 5 months of moderate intensity aerobic exercise training at either 150 or 300 min/week. Since our traditional biomarker results conflict with the current literature and our study is likely not powered to support a true lack of effect, definitive evidence that VO_2_max is a superior biomarker for monitoring aerobic exercise in this specific population will require a randomized controlled study priori powered to assess dose‐dependent changes in traditional biomarkers of health. Our results provide important direction for future research evaluating the effects of aerobic exercise in healthy women at high genetic risk for breast cancer interested in closely monitoring the impact of their prescribed exercise doses, as aerobic exercise in this population may delay the onset of breast cancer and reduce cancer mortality.

## CONFLICT OF INTEREST

The authors have stated explicitly that there are no conflicts of interest in connection with this article.

## ETHICAL STATEMENT

This study was approved by the University of Pennsylvania Institutional Review Board, and written consent was obtained prior to participation by all participants and sent to their respective physicians.

## AUTHOR CONTRIBUTIONS

All authors had full access to the data in the study and take responsibility for the integrity of the data and the accuracy of the data analysis. *Conceptualization*, K.H.S.; *Methodology*, K.H.S.; *Investigation*, K.M.S.; *Formal Analysis*, C.E. and S.Z.; *Resources*, K.H.S.; *Writing‐Original Draft*, C.E., S.Z. and K.M.S.; *Writing‐Review and Editing*, C.E., S.Z., J.C.T. and K.M.S.; *Data Visualization*, C.E.; *Supervision*, K.M.S.; *Funding Acquisition*, J.C.T. and K.H.S.; *Data Curation*, C.E. and K.M.S.

## Data Availability

The data that support the findings of this study are available from the corresponding author upon reasonable request.
